# Supplementary material for: Study protocol: Examining sexual and reproductive health literacy in Mexican American young women using a positive deviance approach
Source: PLoS One. 2024 May 23;19(5):e0303974. doi: 10.1371/journal.pone.0303974 (PMC11115210; doi:10.1371/journal.pone.0303974)
Supplement: Attachment — Submitted filename: Study protocol.pdf [file pone.0303974.s001.pdf]

Dear authors,

Thank you for your efforts in studying marginalized groups and individuals who speak languages other than English, such as Spanish. There is still much more work to be done beyond solely including English speakers. Your efforts are appreciated.

Every research endeavor demands a significant investment of effort; therefore, maximizing its validity whenever possible is essential. Two key suggestions to enhance validity include referencing the (1) mixed-method research protocol outlined by Venkatesh, V., Brown, S.A., and Sullivan, Y.W. (2016) in the "Guidelines for Conducting Mixed-methods Research: An Extension and Illustration," Journal of the AIS (17:7), 435-495, and (2) utilizing the forward-backward translation technique proposed by Guillemin F, Bombardier C, and Beaton D. in "Crosscultural adaptation of health-related quality of life measures: literature review and proposed guidelines," J Clin Epidemiol. 1993;46:1417-1432.

Other comments are as follows:

1. In lines 36-38, to clarify the role of the Community Advisory Committee (CAC) in this study, it may be beneficial to explicitly state that the CAC will be formed to provide community-engaged guidance and support for the recruitment of participants.
2. In line 87, does "successful" refer to the criteria detailed in lines 212-215? If so, it would enhance clarity for readers to provide examples of what is meant by "success" immediately. This is important as "success" is one of your main definitions.
3. Line 90-94: Are these two sentences the same?
4. In line 128-132, according to Figure 1, "environmental" is one of the variables, but it is omitted in Aim 1. Is this intentional, and if so, why?
5. In line 143, to ensure the validity of the mixed method protocol, please consider incorporating the mixed-method protocol suggested by Venkatesh, V., Brown, S.A., and Sullivan, Y.W. (2016) in the "Guidelines for Conducting Mixed-methods Research: An Extension and Illustration," Journal of the AIS (17:7), 435-495. For instance, questions regarding the property of mixed-method research, such as whether quantitative and qualitative data collection occur sequentially or concurrently, are not explicitly addressed.
6. In line 161-164, according to Figure 1, "environmental" is one of the variables, but it is omitted in Aim 1. Is this intentional, and if so, why?
7. In line 188, are "medical providers" and "healthcare providers" referring to the same group of individuals? This question arises because in Figure 2, "medical providers" are not included.
8. In line 264, could you please clarify what occurs if participants choose to withdraw after completing the interview?
9. Line 269: What is HIPAA? Do you mean the Health Insurance Portability and Accountability Act? Please clarify the full term for readers who may not be familiar with the abbreviation.
10. Line 295: Is it 5 or 6 options? Please double-check.
11. In line 302, why are only 3 questions out of the 5 items from the Centrality of Religiosity Scale (CRS-5) being used to measure religiosity? Please clarify

12. In line 307, could you explain how the validity of the translation is ensured, particularly considering the utilization of forward and back translation techniques?
13. In lines 328 and 329, please consider including forward and back translation techniques to ensure validity.
14. Line 364: "RYMAW," is this a typo?
